# B-Lines Scores Derived From Lung Ultrasound Provide Accurate Prediction of Extravascular Lung Water Index: An Observational Study in Critically Ill Patients

**DOI:** 10.1177/0885066620967655

**Published:** 2020-11-05

**Authors:** Ulrich Mayr, Marina Lukas, Livia Habenicht, Johannes Wiessner, Markus Heilmaier, Jörg Ulrich, Sebastian Rasch, Roland M. Schmid, Tobias Lahmer, Wolfgang Huber, Alexander Herner

**Affiliations:** 1 Klinik und Poliklinik für Innere Medizin II, Klinikum rechts der Isar, Technische Universität München, München, Germany; † Wolfgang Huber deceased

**Keywords:** lung ultrasound, B-lines, pulmonary edema, extravascular lung water index (EVLWI), pulmonary vascular permeability index (PVPI), transpulmonary thermodilution (TPTD), acute respiratory distress syndrome (ARDS), intensive care unit (ICU)

## Abstract

**Introduction:**

Visualization of B-lines via lung ultrasound provides a non-invasive estimation of pulmonary hydration. Extravascular lung water index (EVLWI) and pulmonary vascular permeability index (PVPI) assessed by transpulmonary thermodilution (TPTD) represent the most validated parameters of lung water and alveolocapillary permeability, but measurement is invasive and expensive. This study aimed to compare the correlations of B-lines scores from extensive 28-sector and simplified 4-sector chest scan with EVLWI and PVPI derived from TPTD in the setting of intensive care unit (primary endpoint).

**Methods:**

We performed scoring of 28-sector and 4-sector B-Lines in 50 critically ill patients. TPTD was carried out with the PiCCO-2-device (Pulsion Medical Systems SE, Maquet Getinge Group). Median time exposure for ultrasound procedure was 12 minutes for 28-sector and 4 minutes for 4-sector scan.

**Results:**

Primarily, we found close correlations of 28-sector as well as 4-sector B-Lines scores with EVLWI (R^2^ = 0.895 vs. R^2^ = 0.880) and PVPI (R^2^ = 0.760 vs. R^2^ = 0.742). Both B-lines scores showed high accuracy to identify patients with specific levels of EVLWI and PVPI. The extensive 28-sector B-lines score revealed a moderate advantage compared to simplified 4-sector scan in detecting a normal EVLWI ≤ 7 (28-sector scan: sensitivity = 81.8%, specificity = 94.9%, AUC = 0.939 versus 4-sector scan: sensitivity = 81.8%, specificity = 82.1%, AUC = 0.902). Both protocols were approximately equivalent in prediction of lung edema with EVLWI ≥ 10 (28-sector scan: sensitivity = 88.9%, specificity = 95.7%, AUC = 0.977 versus 4-sector scan: sensitivity = 81.5%, specificity = 91.3%, AUC = 0.958) or severe pulmonary edema with EVLWI ≥ 15 (28-sector scan: sensitivity = 91.7%, specificity = 97.4%, AUC = 0.995 versus 4-sector scan: sensitivity = 91.7%, specificity = 92.1%, AUC = 0.978). As secondary endpoints, our evaluations resulted in significant associations of 28-sector as well as simplified 4-sector B-Lines score with parameters of respiratory function.

**Conclusion:**

Both B-line protocols provide accurate non-invasive evaluation of lung water in critically ill patients. The 28-sector scan offers a marginal advantage in prediction of pulmonary edema, but needs substantially more time than 4-sector scan.

## Introduction

Pulmonary edema is a highly-frequent disorder in critically ill patients and a well-characterized hallmark of acute respiratory distress syndrome (ARDS).^1^ It may be triggered by fluid overload, increased pulmonary capillary permeability or congestive heart failure.^2,3^ The pathological accumulation of lung water is related to impaired prognosis. In particular, inappropriate initial therapy is associated with increased mortality.^4^ Consequently, non-invasive methods with high reliability and validity for early identification of pulmonary edema offer diagnostic advantages as well as therapeutic options to prevent progression of lung failure.^5^

The amount of fluid accumulated in alveolar, interstitial and intracellular compartments is summarized as extravascular lung water (EVLW).^6^ High EVLW is the objectifiable result of increased hydrostatic pressure and capillary permeability. EVLW is typically elevated in syndromes like ARDS or sepsis.^7,8^ In current intensive care setting, transpulmonary thermodilution (TPTD) provides a practical method to assess hemodynamic as well as lung parameters at the bedside.^9^ Previous studies outlined that indexation to predicted body weight (EVLWI) was well correlated to oxygenation parameters and mortality in patients with acute lung injury (ALI) or ARDS.^9,10^ In addition to EVLWI, TPTD offers an assessment of pulmonary vascular permeability index (PVPI). More precisely, PVPI reflects the amount of extravascular pulmonary water in proportion to the pulmonary blood volume.^11,12^ Numerous previous studies evaluated the high prognostic value of EVLWI and PVPI in critically ill patients.^7,13,14^ In the specific setting of ARDS, both of them were rated as independent predictors of mortality.^15–17^ Because of invasiveness and limited availability of TPTD however, EVLWI and PVPI are not incorporated in the Berlin definition of ARDS so far.^18,19^

Early, non-invasive and easily-applicable detection of pulmonary edema is still an ambitious and attractive goal.^5,20^ Considering the drawbacks of clinical and radiological techniques, the possibility of fast and accurate lung ultrasound at the bedside has become increasingly popular in intensive care medicine.^21^ Sonographic visualization of B-lines—originally termed as *comet-tail artifacts* arising vertically from the hyperechoic pleural line—represents a promising alternative for assessment of lung water.^22^ Scoring of B-lines is typically performed by their summation from different intercostal spaces.^20^ Most commonly recommended, an extensive 28-sector protocol of the antero-lateral chest is used for evaluation of quantitative B-lines score.^23,24^ Nevertheless, previous studies even described a strong positive correlation of EVLWI with simplified B-lines scores derived from limited 4-sector or 8-sector chest scans.^25,26^ However, comparative analyses of different scanning protocols are rare so far.

Timely diagnosis of pulmonary edema is of vital importance for rapid detection and optimized treatment of respiratory dysfunction in patients transferred to intensive care unit (ICU). EVLWI and PVPI are the gold standard for quantification of lung water and permeability of alveolocapillary barrier. The primary aim of the present study was to compare correlations of B-lines scores derived from 28-sector and simplified 4-sector scan with lung water parameters assessed by TPTD in critically ill patients.

## Methods

### Study Design

This observational study was approved by the institutional review board (Ethikkommission Technische Universität München; Fakultät für Medizin; Project number 5384/12). Informed consent was obtained by patients or their representatives. Between January 2017 and May 2018, we screened a total of 78 patients on admission to our ten-bed university hospital ICU with hemodynamic monitoring via TPTD for feasibility of transthoracic ultrasound with quantification of B-lines. TPTD was performed irrespective of the study based on the indication made by the treating ICU physician. Due to influences on lung ultrasound and B-lines score, patients with visible pleural effusion at scanning-regions were excluded (n = 13). Furthermore, we excluded all patients with proven pulmonary vascular occlusion (n = 2) or major 1-sided pathologies i.e. large pleural effusion (n = 5), pneumothorax (n = 2), thoracic drainage (n = 1), extended atelectasis (n = 2), tumorous lesion (n = 2) or former lung resection (n = 1). Finally, we analyzed a total of 50 critically ill patients in the present study.

## Techniques

### Lung Ultrasound and Quantification of B-Lines Scores

**Figure 1.**
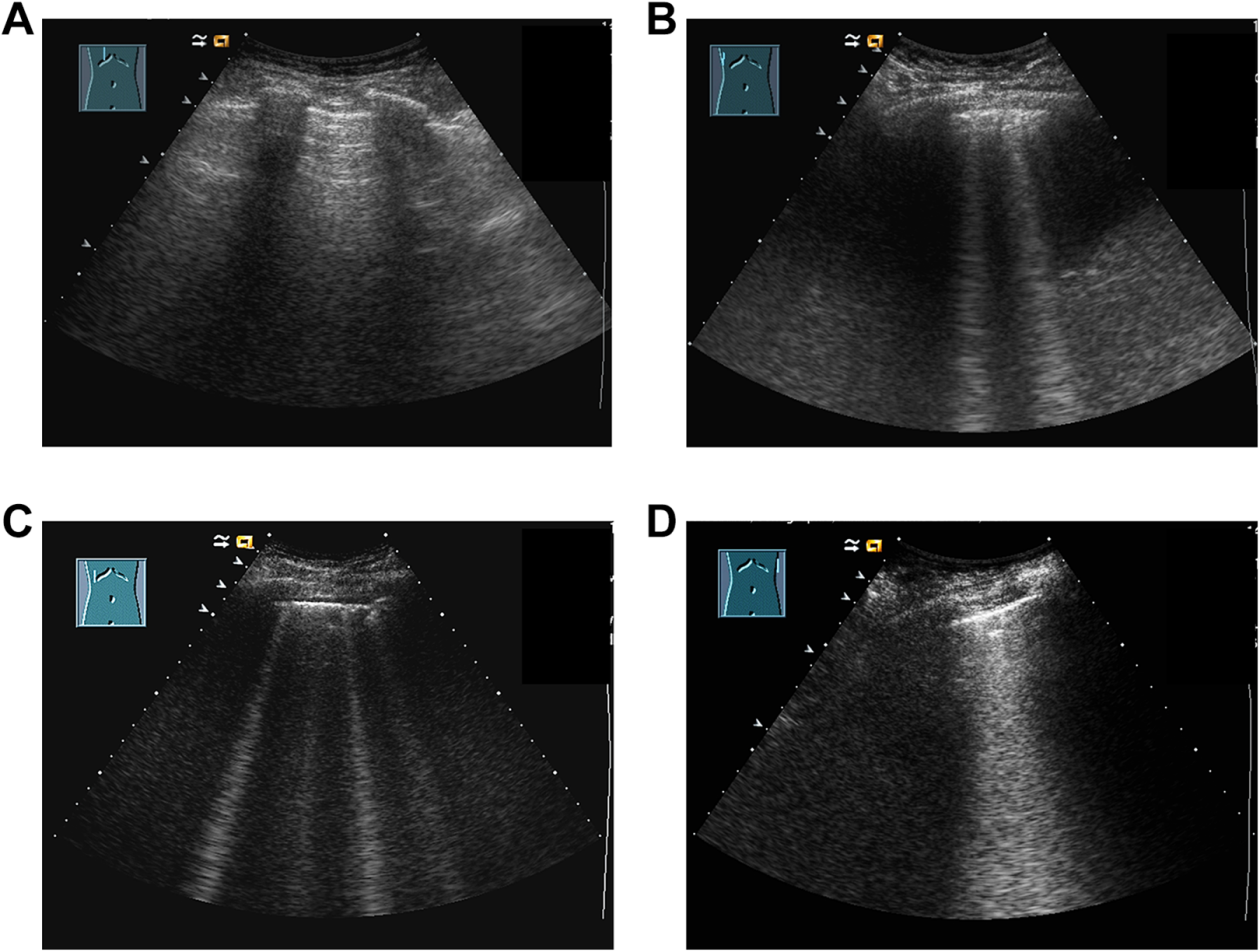
Exemplary pictures of B-lines-visualization of different intercostal spaces (ICS): (A) Absent B-lines and predominant horizontal A-lines, (B) 2 B-lines / ICS, (C) 4 B-lines / ICS, (D) confluent B-lines 50-75% ICS.

Transthoracic ultrasound was accomplished non-invasively at the bedside in supine position on the day of the ICU-admission. We examined B-lines shortly after placement of TPTD-catheters before starting initial TPTD-assessment. All analyses were performed by a single physician with 8 years of institutional experience in the field of ultrasound (U. M.). This investigator was blinded to individual medical history, laboratory data, respiratory function and ventilatory parameters of examined patients. We used the mobile ultrasound scanner ACUSON X300 (Siemens Healthcare GmbH, Erlangen, Germany) and a convex 3.5 Mhz transducer. Figure 1 shows exemplary pictures of B-lines-visualization from transthoracic ultrasound.

**Figure 2.**
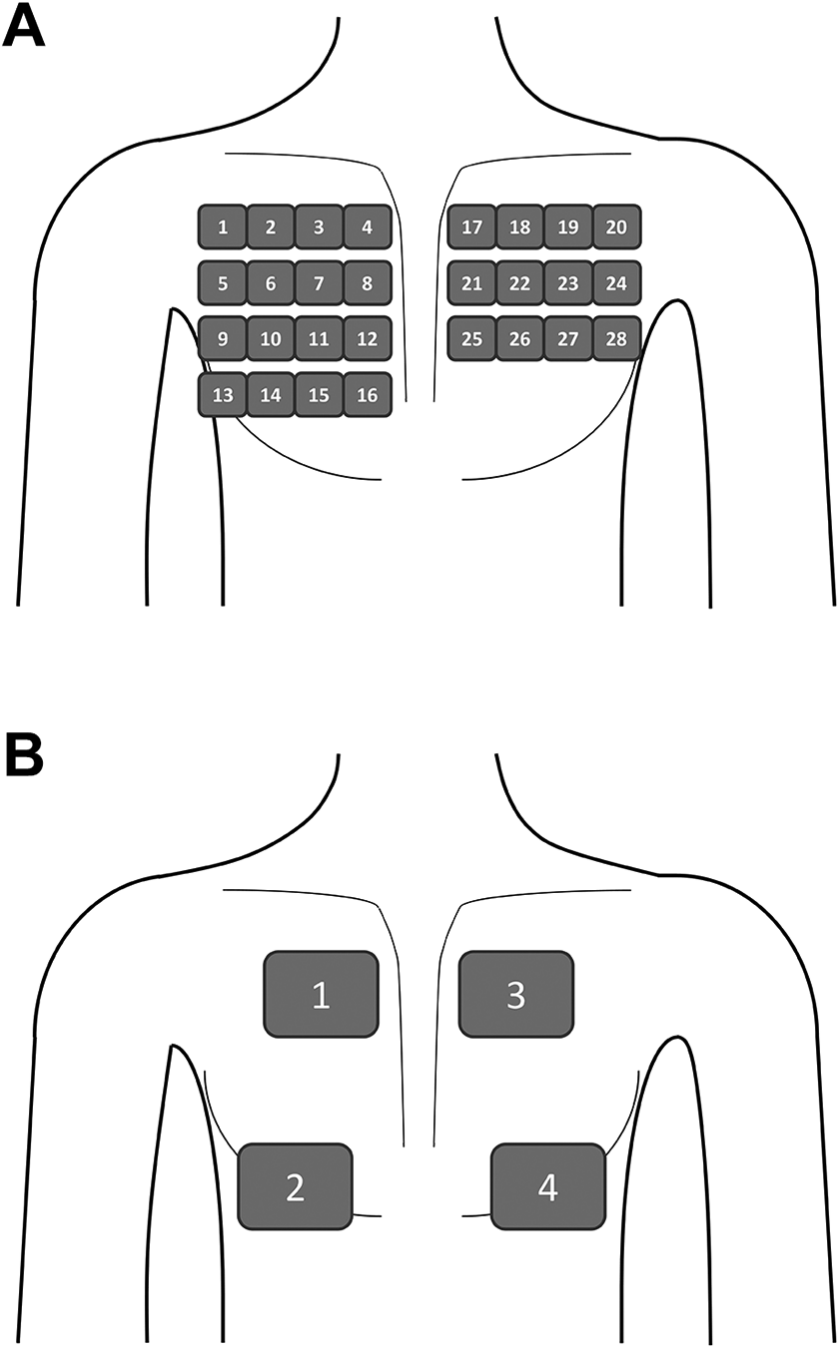
Schematic illustration of the different scanning regions used for 28-7 sector protocol (A) as well as 4-sector chest scan (B).

For accurate 28-sector scan of the antero-lateral chest, we used the extensive protocol as described earlier.^23,24^ 28-sector B-lines score (28s-BL) was quantified by summation of B-lines from all intercostal spaces as illustrated in supplemental file 1. Simplified 4-sector scan and corresponding scoring of 4-sector B-lines (4s-BL) was done as shown in supplemental file 2 and described by Enghard et al.^26^ A scheme of the different scanning regions for 28-sector protocol as well as 4-sector chest scan is depicted in Figure 2. The examining physician performed the ultrasound scan and made prints of each scanned intercostal region. A further physician of our ICU (A. H.)—blinded to the ultrasound procedure and results of TPTD—analyzed the printed screenshots using exactly the same scoring system. Finally, the results of the physician performing the ultrasound and the one examining the prints were averaged to the 28s-BL and 4s-BL score evaluated in this study.

## Hemodynamic Monitoring

All patients were under hemodynamic monitoring via TPTD with the PiCCO-2-device (Pulsion® Medical Systems SE, Maquet Getinge Group) as described previously^27,28^: A 5 Fr thermistor-tipped arterial line (Pulsiocath, Pulsion® Medical Systems, Maquet Getinge Group) inserted through a femoral artery and a hemodynamic monitor (PiCCO-2, Pulsion® Medical Systems, Maquet Getinge Group) served to derive and analyze the thermodilution curve after injection of a cold indicator bolus (15-20 mL of saline cooled down to 4° C) through a jugular central venous catheter. Measurements were done in triplicate, averaged and automatically indexed according to manufacturer´s recommendations to assess EVLWI, PVPI, global end-diastolic volume index (GEDVI) and cardiac index (CI). Central venous pressure (CVP) was measured via the central venous catheter at end-expiration.

## Thresholds for Stratification of EVLWI and PVPI

According to earlier studies we distinguished 3 different categories of EVLWI: normal range without pulmonary edema was defined as EVLWI ≤ 7, while pathological accumulation of lung water was determined with an EVLWI ≥ 8.^7,29^ A cut-off of EVLWI ≥ 10 was chosen for lung edema and patients with EVLWI ≥ 15 were rated as severe edema.^7,30–32^

Analogously, we categorized patients in dependence of PVPI: Normal permeability was assumed in case of PVPI < 2, while a cut-off of PVPI ≥ 3 was chosen to define severely increased vascular permeability.^11,33^

## Ventilator Setting and Respiratory Function

Patients with spontaneous breathing received a demand-based application of oxygen. Mechanical ventilation was performed using the routine ventilator device EVITA XL of our ICU (Dräger, Lübeck, Germany). Parameters were set according to current ARDSNet recommendations, especially regarding positive end-expiratory pressure (PEEP).^34^ Ventilator setting was based on medical assessment by the treating ICU physician irrespective of the study. The EVITA XL ventilator continuously monitored levels of airway pressures and corresponding volumes. Ventilatory parameters such as PEEP, mean airway pressure (P_mean_), dynamic respiratory system compliance (C_dyn_) and fraction of inspired oxygen (F_i_O_2_) were recorded immediately after lung ultrasound. P_a_O_2_ and p_a_CO_2_ were derived from a fully-automatic blood gas analysis device (Rapid Point 400, Siemens Healthcare Diagnostic GmbH, Eschborn, Germany). Blood gas analysis and ventilatory parameters were used for calculation of Horowitz-index (p_a_O_2_/F_i_O_2_) and Oxygenation Index (OI = F_i_O_2_*mean airway pressure*100/p_a_O_2_).^35^

## Data Collection

Clinical and laboratory parameters for the calculation of APACHE II- and SOFA-score were recorded on the day of ultrasound and TPTD. Ultrasound examination was done immediately before TPTD. Ventilator settings, respiratory and hemodynamic profiles were recorded immediately after ultrasound and TPTD.

## Statistical Analysis and Primary Endpoint

For primary outcome analysis we correlated 28s-BL as well as 4s-BL with EVLWI and PVPI. All correlations were done using Spearman´s correlation coefficient r and linear regressions using the coefficient R^2^. Bland-Altman-plots were performed for EVLWI and PVPI to check for possible biases. Continuous variables are expressed as median and interquartile range (IQR), categorical variables are expressed as percentages. Receiver-operating-characteristic curves (ROC) were used to specify the diagnostic potential of 28s-BL and 4s-BL for prediction of certain levels of EVLWI and PVPI via area under curve (AUC). Appropriate cut-offs were identified by highest combined sensitivity and specificity using Youden‘s index. All analyses and graphs were generated using GraphPad Prism 8.0 (GraphPad Software, La Jolla, CA, USA). Significance was assumed at a p-value < 0.05.

## Results

### Patients’ Baseline Characteristics

Patients’ baseline characteristics and clinical scores are presented in Table 1.

**Table 1.** Patients Baseline Characteristics and Clinical Scores.

**Patients characteristics**	
**Male sex, n/total (%)**	32/50 (64%)
**Age, years**	65 (55-72)
**Body weight, kg**	82 (74-90)
**Body height, cm**	175 (168-180)
**APACHE II**	20 (16-26)
**SOFA**	9 (7-13)
**Admission diagnoses, n/total (%)**	Pneumonia/ARDS 17/50 (34%)
	Sepsis/MOV 10/50 (20%)
	Liver cirrhosis 10/50 (20%)
	Pancreatitis 7/50 (14%)
	Others 6/50 (12%)
**Mode of ventilation, n/total (%)**	Spontaneous breathing 8/50 (16%)
	Pressure-supported 21/50 (42%)
	Pressure-controlled 21/50 (42%)
**PEEP, cmH_2_O**	8 (6-10), Min-Max: 5-14
**F_i_O_2_, %**	40 (30-50), Min-Max: 21-90
**P_mean_, cmH_2_O**	13 (10-15), Min-Max: 6-22
**p_a_CO_2_, mmHg**	38 (33-45), Min-Max: 26-61
**C_dyn_, mL/cmH_2_O**	42 (37-56), Min-Max: 13-121
**p_a_O_2_/F_i_O_2_, mmHg**	219 (177-287), Min-Max: 75-448
**OI**	6.1 (3.7-8.7), Min-Max: 1.3-23.3
**EVLWI, mL/kg**	10 (8-15), Min-Max: 5-27
**PVPI**	1.7 (1.2-2.1), Min-Max: 0.8-5.8
**GEDVI, mL/m^2^**	767 (690-900), Min-Max: 505-1696
**CVP, mmHg**	16 (11-19), Min-Max: 5-36
**CI, L/min/m^2^**	3.8 (3.1-4.7), Min-Max: 2.1-5.8
**28s-BL**	17 (7-26), Min-Max: 3-46
**Subdivision of 28s-BL, n/total (%)**	≤ 5 (absent): 7/50 (14%)
	6-15 (mild degree): 17/50 (34%)
	16-30 (moderate degree): 17/50 (34%)
	> 30 (severe degree): 9/50 (18%)
**4s-BL**	10 (4-16), Min-Max: 2-28

APACHE: Acute physiology and chronic health evaluation; SOFA: Sequential organ failure assessment; PEEP: Positive end-expiratory pressure; F_i_O_2_: Fraction of inspired oxygen; P_mean_: Mean airway pressure; p_a_CO_2_: Arterial partial pressure of carbon dioxide; C_dyn_: Dynamic respiratory system compliance; p_a_O_2_: Arterial partial pressure of oxygen; OI: Oxygenation index; EVLWI: Extravascular lung water index; PVPI: Pulmonary vascular permeability index; GEDVI: Global end-diastolic volume index; CVP: Central venous pressure; CI: Cardiac index; 28s-BL 28-sector B-lines; 4s-BL 4-sector B-lines.

We performed scoring of 28s-BL and 4s-BL in a total of 50 patients (18 female and 32 male patients). APACHE- and SOFA-scores are compatible with critical illness of our population. 84% of all patients were mechanically ventilated and 16% were spontaneously breathing. Ventilator setting remained unchanged during study measurements and was based on the decision of the treating physician.

Extensive 28-sector protocol as illustrated in supplemental file 1 and Figure 2A was used for assessment of 28s-BL (23,24). Median examination time for 28-sector scan was 12 (10-14) minutes. Our analyses resulted in a median 28s-BL of 17 (7-26) in all 50 patients. According to the recommendation by Picano and Pellikka,^20^ patients were subdivided into 4 different grades of lung water depending on summed B-lines: 7 patients were categorized as “absent” lung water (28s-BL ≤ 5), 17 patients as “mild degree” (28s-BL 6-15), 17 patients as “moderate degree” (28s-BL 16-30) and 9 patients were classified as “severe degree” of lung water (28s-BL ≥ 30).

Simplified 4-sector scan was performed as depicted in supplemental file 2 and Figure 2B.^26^ The corresponding scoring resulted in a median 4s-BL of 10 (4-16) in all 50 patients. Median time exposure for limited 4-sector scan was 4 (3-7) minutes.

## Correlations and Regression Plots

**Figure 3.**
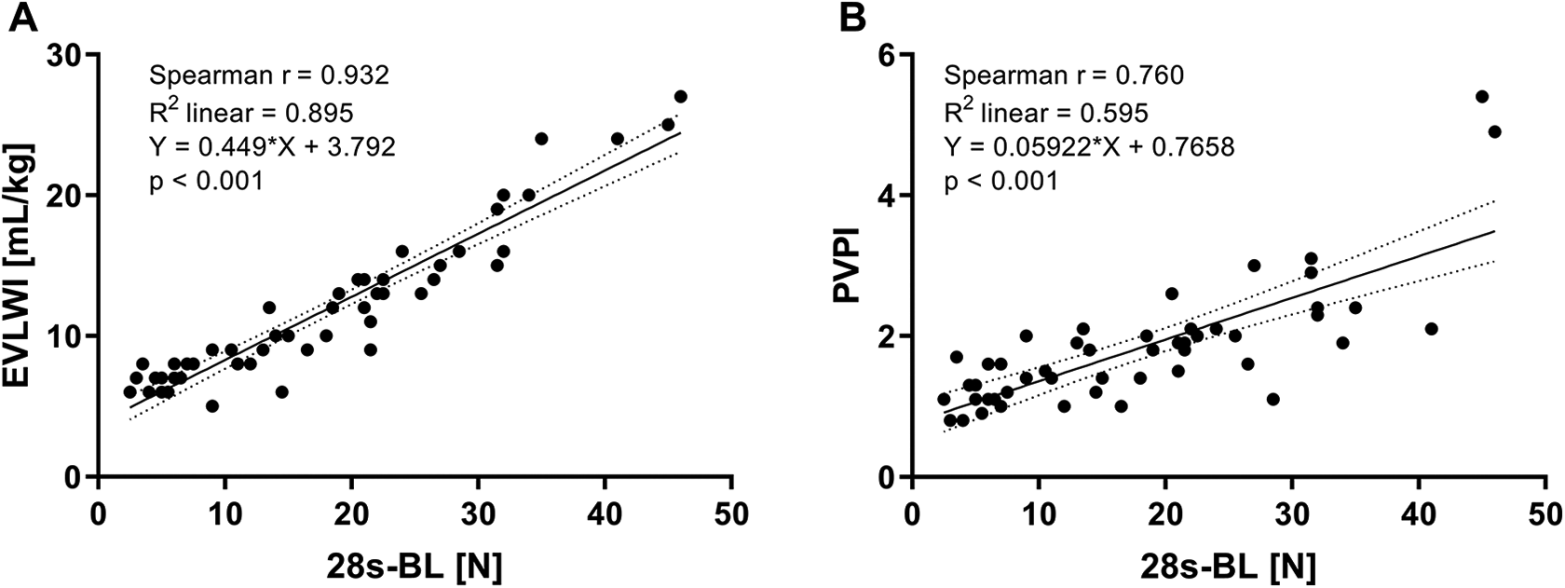
Correlations of summed 28-sector B-lines score (28s-BL) with: (A) Extravascular lung water index (EVLWI), (B) Pulmonary vascular permeability index (PVPI).

Analyses for extensive 28s-BL score with lung water indices are illustrated in Figure 3. In detail, our results revealed a significant association between 28s-BL and EVLWI ([A], r = 0.932, R^2^ = 0.895, p < 0.001) as well as PVPI ([B], r = 0.760, R^2^ = 0.595, p < 0.001). As shown in Table 2, we found statistically significant correlations with p_a_O_2_/F_i_O_2_ (p < 0.001), OI (p < 0.001) and C_dyn_ (p < 0.001). Our analyses resulted in weak but still significant associations of 28s-BL with p_a_CO_2_ (p = 0.036) and CVP (p = 0.024), but not with the preload parameter GEDVI (p = 0.170), nor with CI (p = 0.227). Finally, 28s-BL correlated closely with simplified 4s-BL (p < 0.001).

**Table 2.** Correlations and Linear Regressions for 28-Sector B-Lines Score (28s-BL).

**Correlation coefficient and linear regressions for 28s-BL with various respiratory and hemodynamic parameters**			
	**Spearmans** **coefficient r**	**Linear regression** **R^2^**	**p-value**
**p_a_O_2_/F_i_O_2_**	-0.521	0.326	<0.001
**OI**	0.572	0.444	<0.001
**C_dyn_**	-0.595	0.310	<0.001
**p_a_CO_2_**	0.297	0.047	0.036
**CVP**	0.316	0.136	0.024
**GEDVI**	0.197	0.060	0.170
**CI**	0.174	0.030	0.227
**4s-BL**	0.946	0.908	<0.001

OI: Oxygenation index; Cdyn: Dynamic respiratory system compliance; CVP: Central venous pressure; GEDVI: Global end-diastolic volume index; CI: Cardiac index; 4s-BL: 4-sector B-lines score.

**Figure 4.**
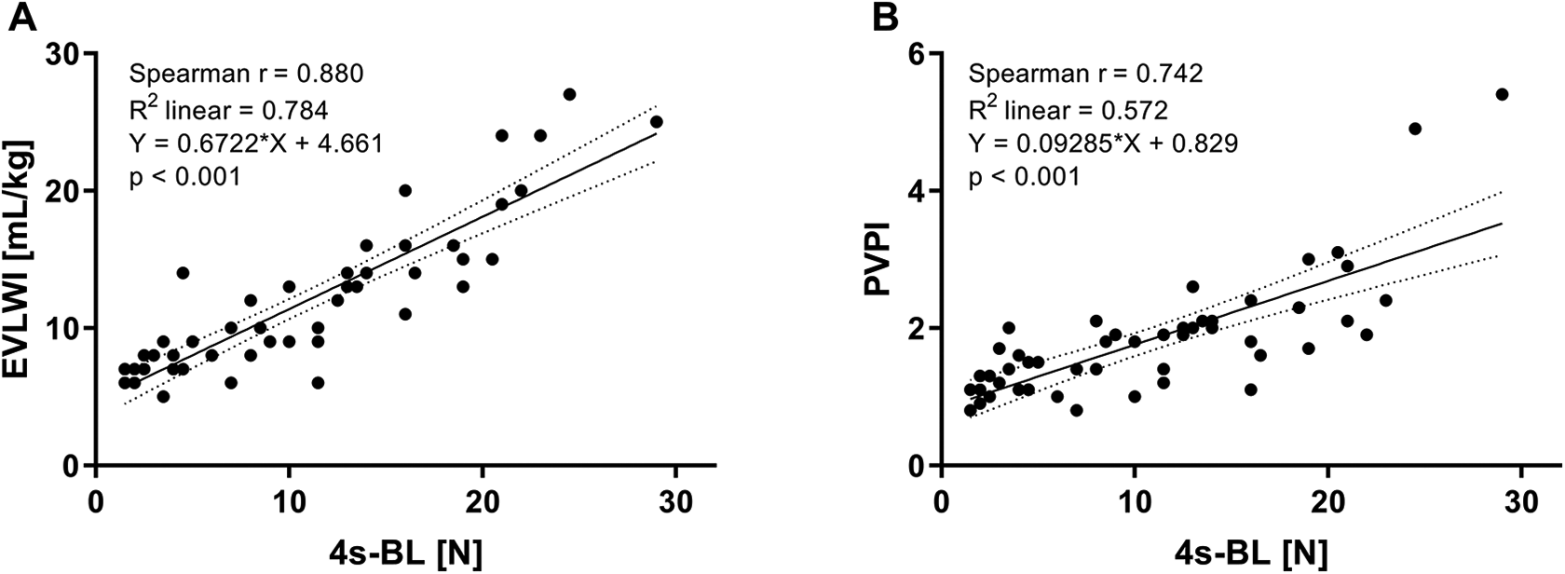
Correlations of simplified 4-sector B-lines score (4s-BL) with: (A) Extravascular lung water index (EVLWI), (B) Pulmonary vascular permeability index (PVPI).

Analogously, regression plots for simplified 4s-BL score are depicted in Figure 4. Positive correlations with EVLWI ([A], r = 0.880, R^2^ = 0.784, p < 0.001) and PVPI ([B], r = 0.742, R^2^ = 0.572, p < 0.001) were high, but a little bit lower as compared to associations of 28s-BL mentioned earlier. We also found statistically significant associations of 4s-BL with p_a_O_2_/F_i_O_2_ (p < 0.001), OI (p < 0.001), C_dyn_ (p < 0.001) and CVP (p = 0.039), but not with p_a_CO_2_ (p = 0.078), GEDVI (p = 0.178) or CI (p = 0.120) (Table 3).

**Table 3.** Correlations and Linear Regressions for 4-Sector B-Lines Score (4s-BL).

**Correlation coefficient and linear regressions for 4s-BL with various respiratory and hemodynamic parameters**			
	**Spearmans** **coefficient r**	**Linear regression** **R^2^**	**p-value**
**p_a_O_2_/F_i_O_2_**	-0.521	0.292	<0.001
**OI**	0.544	0.367	<0.001
**C_dyn_**	-0.581	0.250	<0.001
**p_a_CO_2_**	0.252	0.037	0.078
**CVP**	0.293	0.109	0.039
**GEDVI**	0.194	0.020	0.178
**CI**	0.223	0.044	0.120

OI: Oxygenation index; C_dyn_: Dynamic respiratory system compliance; CVP: Central venous pressure; GEDVI: Global end-diastolic volume index; CI: Cardiac index.

## Bland-Altman plots

Concerning the significant associations of both 28s-BL as well as 4s-BL with lung water indices assessed by TPTD, we additionally performed Bland-Altman plots to address for any potential biases: We characterized the indices directly assessed from TPTD as EVLWI_TPTD_ and PVPI_TPTD_. The corresponding indices calculated from the correlation plots with B-lines scores were labeled as EVLWI_28s-BL_ and PVPI_28s-BL_ as well as EVLWI_4s-BL_ and PVPI_4s-BL_, respectively.

As shown in supplemental file 3, a plot of EVLWI_TPTD_ vs. EVLWI_28s-BL_ resulted in a low bias of 0.062 ([A], SD 1.8, 95% limits −3.4 to 3.5). A plot of PVPI_TPTD_ vs. PVPI_28s-BL_ also revealed a low bias of -0.006 ([B] SD 0.6, 95% limits −1.1 to 1.1).

Analogously, Bland-Altman plots for the indices calculated from 4s-BL scores are illustrated in supplemental file 4: We found a low bias of -0.0003 for EVLWI_TPTD_ vs. EVLWI_4s-BL_ ([A] SD 2.5, 95% limits −5.0 to 5.0) and a low bias of -0.0001 for PVPI_TPTD_ vs. PVPI_4s-BL_ ([B] SD 0.6, 95% limits −1.1 to 1.1).

Supplemental file 3: Bland-Altman plots of lung water indices assessed by transpulmonary thermodilution (TPTD) vs. corresponding indices calculated from correlation plots with 28-sector B-lines score (28s-BL): [A] Difference EVLWI_TPTD_- EVLWI_28s-BL_ vs. Average, [B] Difference PVPI_TPTD_-PVPI_28s-BL_ vs. Average

Supplemental file 4: Bland-Altman plots of lung water indices assessed by transpulmonary thermodilution (TPTD) vs. corresponding indices calculated from correlation plots with 4-sector B-lines score (4s-BL): [A] Difference EVLWI_TPTD_- EVLWI_4s-BL_ vs. Average, [B] Difference PVPI_TPTD_-PVPI_4s-BL_ vs. Average

## Receiver Operating Characteristic Curves

**Figure 5.**
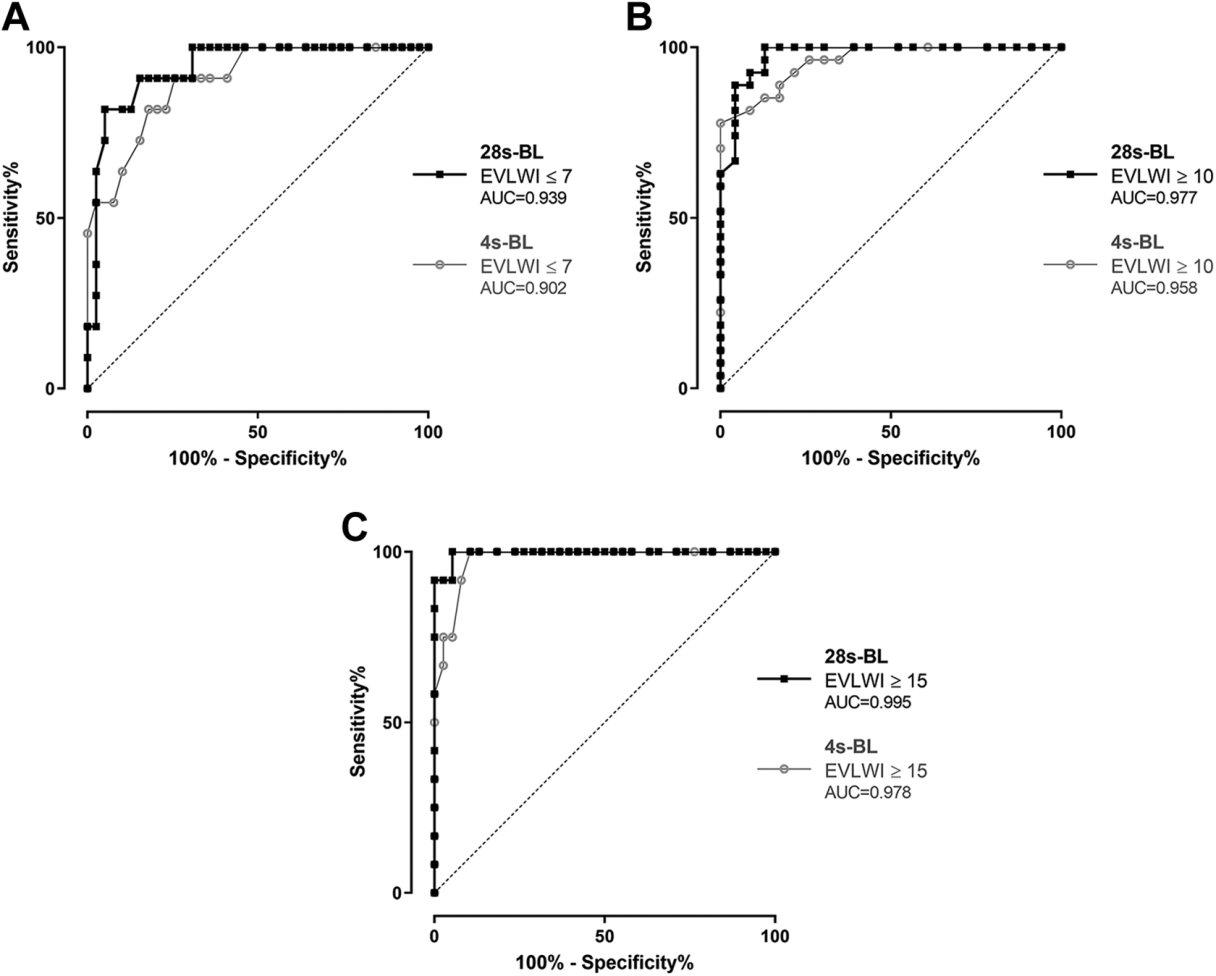
Receiver operating characteristic (ROC) curves analyzing the diagnostic potential of 28-sector B-lines (28s-BL) and 4-sector B-lines score (4s-BL) to identify patients with: (A) EVLWI ≤ 7, (B) EVLWI ≥ 10, (C) EVLWI ≥ 15.

ROC curves were performed to evaluate the diagnostic potential of B-lines scores for prediction of specific levels of EVLWI and PVPI. First of all, we analyzed the potential of both protocols to identify patients with an EVLWI in the normal range ≤ 7 (Figure 5A: A summed 28s-BL score < 7 was associated with a sensitivity of 81.8% and a specificity of 94.9% to predict an EVLWI ≤ 7 (AUC = 0.939). For comparison, a cut-off of 4s-BL < 5 to identify EVLWI ≤ 7 had a sensitivity of 81.8% and a specificity of 82.1% (AUC = 0.902). Furthermore, we analyzed the potential for prediction of lung edema with EVLWI ≥ 10 (Figure 5B): We found a sensitivity of 88.9% and a specificity of 95.7% if 28s-BL was ≥ 17 (AUC = 0.977), compared to a sensitivity of 81.5% and a specificity of 91.3% if 4s-BL was ≥ 11 (AUC = 0.958). Additional ROC analyses were done for identification of severe lung edema with EVLWI ≥ 15 (Figure 5C): Our tests showed a sensitivity of 91.7% and a specificity of 97.4% if 28s-BL was ≥ 26 (AUC = 0.995). A simplified 4s-BL ≥ 15 resulted in a sensitivity of 91.7% and specificity of 92.1% to identify patients with an EVLWI ≥ 15 (AUC = 0.978).

**Figure 6.**
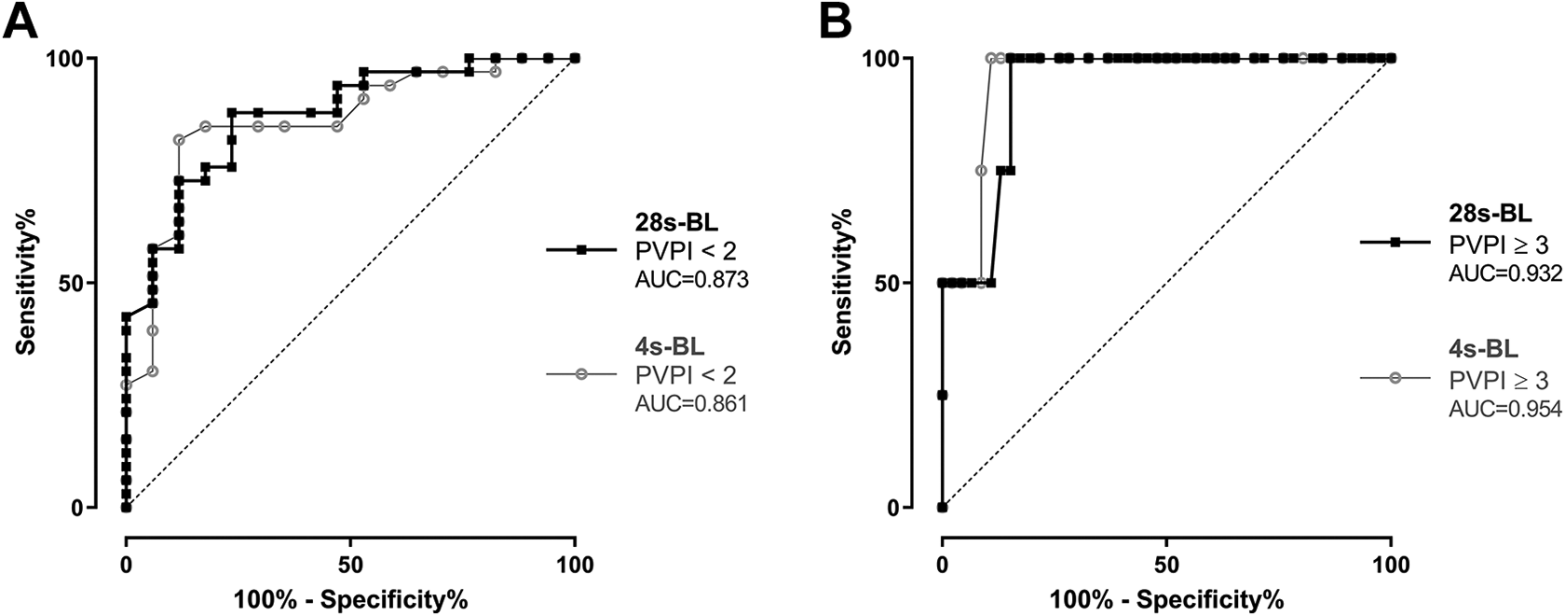
Receiver operating characteristic (ROC) curves analyzing the diagnostic potential of 28-sector B-lines (28s-BL) and 4-sector B-lines score (4s-BL) to identify patients with: (A) PVPI < 2, (B) PVPI ≥ 3.

Moreover, we analyzed the potential of both scores for identification of a normal PVPI < 2 (Figure 6A): A 28s-BL score < 18 had a sensitivity of 72.7% and a specificity of 88.2% to predict a PVPI < 2 (AUC = 0.873), compared to a sensitivity of 81.8% and a specificity of 88.2% if 4s-BL was < 12 (AUC = 0.861). Finally, we performed ROC curves to analyse the diagnostic value of B-lines to predict a critically high PVPI ≥ 3 (Figure 6B) and found a sensitivity of 75% and a specificity of 87% if 28s-BL ≥ 30 (AUC = 0.932), compared to a sensitivity of 75% and a specificity of 91.3% if 4s-BL was ≥ 20 (AUC = 0.954). The results of all ROC curves for identifying patients with specific levels of EVLWI and PVPI are summarized in Table 4.

**Table 4.** Diagnostic Potential of Both B-Lines Protocols for Identification of Specific Levels of EVLWI and PVPI.

**Predictive value of 28-sector scan (28s-BL) and 4-sector scan B-lines score (4s-BL)**								
**Stratification of EVLWI and PVPI**	**28s-BL**				**4s-BL**			
	**Sensitivity (%)**	**Specificity (%)**	**Cut-off** **28s-BL**	**AUC**	**Sensitivity (%)**	**Specificity (%)**	**Cut-off** **4s-BL**	**AUC**
**EVLWI ≤ 7 mL/kg**	81.8	94.9	< 7	0.939	81.8	82.1	< 5	0.902
**EVLWI ≥ 8 mL/kg**	84.6	90.9	≥ 10	0.939	74.4	90.9	≥ 8	0.902
**EVLWI ≥ 10 mL/kg**	88.9	95.7	≥ 17	0.977	81.5	91.3	≥ 11	0.958
**EVLWI ≥ 15 mL/kg**	91.7	97.4	≥ 26	0.995	91.7	92.1	≥ 15	0.978
**PVPI < 2**	72.7	88.2	< 18	0.873	81.8	88.2	< 12	0.861
**PVPI ≥ 3**	75.0	87.0	≥ 30	0.932	75.0	91.3	≥ 20	0.954

EVLWI: Extravascular lung water index; PVPI: Pulmonary vascular permeability index; AUC: Area under curve.

## Discussion

The present study illustrates that lung ultrasound is a promising tool for non-invasive assessment of lung water parameters and accurate identification of pulmonary edema at the bedside in critically ill patients.

Primarily, we found a significant correlation of pulmonary B-Lines scores with extravascular lung water index (EVLWI) assessed by transpulmonary thermodilution (TPTD). Strength of correlation was very similar between 28-sector scan (28s-BL, R^2^ = 0.90) and limited 4-sector scan (4s-BL, R^2^ = 0.88). Analogously to EVLWI, both scanning methods displayed a significant association of B-Lines scores with pulmonary vascular permeability index (PVPI). Furthermore, our study revealed a high discriminative ability of B-lines scores in prediction of specific levels of EVLWI and PVPI. In detail, we found a moderate diagnostic advantage of 28s-BL compared to 4s-BL in non-invasive identification of a normal EVLWI ≤ 7. Concerning the prediction of lung edema (EVLWI ≥ 10) and severe pulmonary edema (EVLWI ≥ 15), our analyses resulted in comparably high accuracy in terms of sensitivity and specificity for both 28s-BL as well as 4s-BL protocol.

The present findings are largely in line with previous evaluations: 28s-BL was rated as a useful non-radiologic indicator of lung water and valuable prognostic tool in patients with dyspnea.^22,24^ However, none of these former studies offers a correlation of the extensive 28-sector protocol to lung water parameters derived from TPTD, the current clinical gold standard in diagnosing pulmonary edema.^6^ As opposed to this, B-lines scores with limited scan regions correlated closely with EVLWI assessed by TPTD: Enghard et al. described a markedly stronger correlation of EVLWI with a simplified 4s-BL score (R^2^ = 0.91) compared to x-ray chest (R^2^ = 0.33) in a total of 50 patients.^26^ Another study by Agricola et al. revealed a significant but much lower correlation of 4-sector scan with EVLWI (R^2^ = 0.42).^25^ Moreover, our study reaffirms that lung ultrasound is suitable to estimate the amount of extravascular lung water: Enghard et al. evaluated the accuracy of simplified 4s-BL in diagnosing an elevated EVLWI ≥ 8 and described a sensitivity of 92.1% and a specificity of 91.7% with an area under curve (AUC) of 0.942. Analogously, they found a sensitivity of 92.3% and a specificity of 94.6% with an AUC of 0.964 for 4s-BL to identify patients with severely increased EVLWI, which was comparable to the results of our analyses.^26^

Additionally, the present study offers some interesting secondary findings. Our analyses demonstrate significant associations of lung ultrasound with parameters of respiratory function: Both extended 28s-BL as well as simplified 4s-BL correlated inversely with Horowitz-index (p_a_O_2_/F_i_O_2_) and dynamic respiratory system compliance (C_dyn_). However, strength of correlation with lung function was lower compared to the high correlation of B-lines with EVLWI. This finding is in parallel with the weaker association of lung ultrasound with p_a_O_2_/F_i_O_2_ described earlier.^26^ Taken into account that numerous variables are contributing to gas exchange and oxygenation next to lung water, the positive association is still remarkable.^16^ Correlation of B-lines with oxygenation index (OI) was stronger compared to Horowitz-index. According to several studies OI was better in prediction of ARDS-outcome compared to ARDS definitions predominantly based on p_a_O_2_/F_i_O_2._^36–38^ Concerning cardiac preload and output, lung ultrasound showed no correlation with global end-diastolic volume index (GEDVI) or cardiac index (CI) assessed by TPTD. In contrast to previous evaluations,^26^ we found a very weak but still significant correlation of 28s-BL and 4s-BL with central venous pressure (CVP). As CVP varies considerably depending on ventilator setting and pressure levels,^39,40^ severity of pulmonary edema and respiratory dysfunction might involve increases of CVP.

The strength of this study is that it underlines the potential of different B-lines scores for precise estimation of EVLWI and PVPI in a challenging population of critically ill patients. TPTD offers accurate assessment of pulmonary edema and increased pulmonary vascular permeability.^7,11,16,41,42^ Nevertheless, TPTD is still an invasive procedure restricted to departments with necessary equipment and associated with a certain time delay due to placement of arterial and venous catheters. Lung ultrasound represents a promising alternative for non-invasive estimation of lung water,^20,22,24–26,43,44^ but consensus on the best protocol for quantification of B-lines is still missing.^45^ The original protocol is based on the 28-sector scan, but most studies correlating B-lines to EVLWI used a simplified 4-sector^25,26^ or 8-sector scan.^17^ The only study so far comparing all different protocols with EVLWI was performed in a total of 89 critically ill patients with sepsis^46^: Pirompanich et al. described a high specificity of 28-sector, 4-sector and 8-sector scan in diagnosing EVLWI ≥ 10, whereas sensitivity was quite low for 4-sector and 8-sector scan. According to our results, both 28-BL as well as 4s-BL showed sufficiently high ability to identify pulmonary edema. The extensive 28s-BL seems to have a moderate diagnostic advantage in prediction of EVLWI. Nevertheless, we have to refer to substantially longer time needed for 28-sector scan (median 12 minutes) in comparison to 4-sector scan (median 4 minutes).

Summarizing, our study emphasizes that lung ultrasound is an accurate method for assessment of lung water and permeability at the bedside. EVLWI has been repeatedly suggested to improve ARDS-Definition.^32,47^ This was well recognized by the experts creating the Berlin-Definition. However, they argued that inclusion of EVLWI was “infeasible based on the lack of availability of transpulmonary thermodilution” in most patients with ARDS.^19^ Consequently, estimation of lung water based on B-lines could be the “missing link” to include EVLWI or its estimate in future definitions of ARDS.

## Limitations

Our study has several limitations. First of all, this is a single centre study with consecutively a limited number of patients. It was performed in a heterogenous population of critically ill patients with various disease entities and consecutively varying modes of ventilation. Subgroup analyses for specific syndromes like sepsis or ARDS are not available. Moreover, procedure of lung ultrasound was accomplished on admission to ICU. There were no further quantifications of B-lines in the course of ICU-treatment. Furthermore, the present study has no information and analyses of patients-outcome or ICU mortality in dependence on specific admission-scores of B-lines. Lung ultrasound was correlated to various respiratory and hemodynamic parameters simultaneously to EVLWI. However, correlation of B-lines to radiological assessment of lung water or echocardiography is not available. A final statement about the superiority of 28s-BL vs. 4s-BL is lacking, as correlation with TPTD and predictive value in ROC-analyses was only slightly higher for 28s-BL whereas time exposure was markedly lower for 4s-BL. We did not include the 8-sector scan protocol in this study, so no conclusion can be made about the role of this approach. Last but not least, there is still no consensus about the thresholds for stratification of EVLWI that were used in this study (regular ≤ 7, manifest lung edema ≥ 10, severe edema ≥ 15).

## Conclusion

Estimation of lung water and identification of pulmonary edema via B-lines is a promising non-invasive tool for frontline critical care clinicians. B-Lines scores derived from 28s-BL reveal higher correlations with EVLWI, but assessment in clinical practice is notably more cumbersome and time-consuming compared to simplified 4s-BL.

## Supplemental Material

Supplementary_file_1 - B-Lines Scores Derived From Lung Ultrasound Provide Accurate Prediction of Extravascular Lung Water Index: An Observational Study in Critically Ill Patients Click here for additional data file. Supplementary_file_1 for B-Lines Scores Derived From Lung Ultrasound Provide Accurate Prediction of Extravascular Lung Water Index: An Observational Study in Critically Ill Patients by Ulrich Mayr, Marina Lukas, Livia Habenicht, Johannes Wiessner, Markus Heilmaier, Jörg Ulrich, Sebastian Rasch, Roland M. Schmid, Tobias Lahmer, Wolfgang Huber and Alexander Herner in Journal of Intensive Care Medicine

Supplementary_file_2 - B-Lines Scores Derived From Lung Ultrasound Provide Accurate Prediction of Extravascular Lung Water Index: An Observational Study in Critically Ill Patients Click here for additional data file. Supplementary_file_2 for B-Lines Scores Derived From Lung Ultrasound Provide Accurate Prediction of Extravascular Lung Water Index: An Observational Study in Critically Ill Patients by Ulrich Mayr, Marina Lukas, Livia Habenicht, Johannes Wiessner, Markus Heilmaier, Jörg Ulrich, Sebastian Rasch, Roland M. Schmid, Tobias Lahmer, Wolfgang Huber and Alexander Herner in Journal of Intensive Care Medicine

Supplementary_file_3 - B-Lines Scores Derived From Lung Ultrasound Provide Accurate Prediction of Extravascular Lung Water Index: An Observational Study in Critically Ill Patients Click here for additional data file. Supplementary_file_3 for B-Lines Scores Derived From Lung Ultrasound Provide Accurate Prediction of Extravascular Lung Water Index: An Observational Study in Critically Ill Patients by Ulrich Mayr, Marina Lukas, Livia Habenicht, Johannes Wiessner, Markus Heilmaier, Jörg Ulrich, Sebastian Rasch, Roland M. Schmid, Tobias Lahmer, Wolfgang Huber and Alexander Herner in Journal of Intensive Care Medicine

Supplementary_file_4 - B-Lines Scores Derived From Lung Ultrasound Provide Accurate Prediction of Extravascular Lung Water Index: An Observational Study in Critically Ill Patients Click here for additional data file. Supplementary_file_4 for B-Lines Scores Derived From Lung Ultrasound Provide Accurate Prediction of Extravascular Lung Water Index: An Observational Study in Critically Ill Patients by Ulrich Mayr, Marina Lukas, Livia Habenicht, Johannes Wiessner, Markus Heilmaier, Jörg Ulrich, Sebastian Rasch, Roland M. Schmid, Tobias Lahmer, Wolfgang Huber and Alexander Herner in Journal of Intensive Care Medicine
