# Supplementary material for: B-Lines Scores Derived From Lung Ultrasound Provide Accurate Prediction of Extravascular Lung Water Index: An Observational Study in Critically Ill Patients
Source: J Intensive Care Med. 2020 Nov 5;37(1):21–31. doi: 10.1177/0885066620967655 (PMC8609506; doi:10.1177/0885066620967655)
Supplement: Supplementary_file_1 - B-Lines Scores Derived From Lung Ultrasound Provide Accurate Prediction of Extravascular Lung Water Index: An Observational Study in Critically Ill Patients [file Supplementary_file_1.pdf]

Supplementary file 1: Protocol of extensive 28-sector chest scan to assess 28-sector B-Lines score (28s-BL) according to earlier studies [23,24].

Extensive 28-sector scan protocol for 28s-BL										
Right side	mid-axillary	anterior-axillary	mid-clavear	para-sternal	ICS	para-sternal	mid-clavear	anterior-axillary	mid-axillary	Left side
					2					
					3					
					4					
					5					

28-BL: 28-sector B-lines score

ICS: Intercostal space
