# Supplementary material for: B-Lines Scores Derived From Lung Ultrasound Provide Accurate Prediction of Extravascular Lung Water Index: An Observational Study in Critically Ill Patients
Source: J Intensive Care Med. 2020 Nov 5;37(1):21–31. doi: 10.1177/0885066620967655 (PMC8609506; doi:10.1177/0885066620967655)
Supplement: Supplementary_file_2 - B-Lines Scores Derived From Lung Ultrasound Provide Accurate Prediction of Extravascular Lung Water Index: An Observational Study in Critically Ill Patients [file Supplementary_file_2.pdf]

Supplementary file 2: Ultrasound scoring system for simplified 4-sector B-lines score (4s-BL) according to Enghard et al. [26].

**Ultrasound scoring system for 4s-BL**	
**Ultrasound finding**	**Score**
**No B line/ICS**	0
**One B line/ICS**	1
**Two B lines/ICS**	2
**Three B lines/ICS**	3
**Four B lines/ICS**	4
**Five B lines/ICS**	5
**Confluent B lines > 50 % ICS**	6
**Confluent B lines > 75 % ICS**	7
**Confluent B lines 100 % ICS**	8

4s-BL: 4-sector B-lines score

ICS: Intercostal space
