# Supplementary figures and images for: B-Lines Scores Derived From Lung Ultrasound Provide Accurate Prediction of Extravascular Lung Water Index: An Observational Study in Critically Ill Patients
Source: J Intensive Care Med. 2020 Nov 5;37(1):21–31. doi: 10.1177/0885066620967655 (PMC8609506; doi:10.1177/0885066620967655)

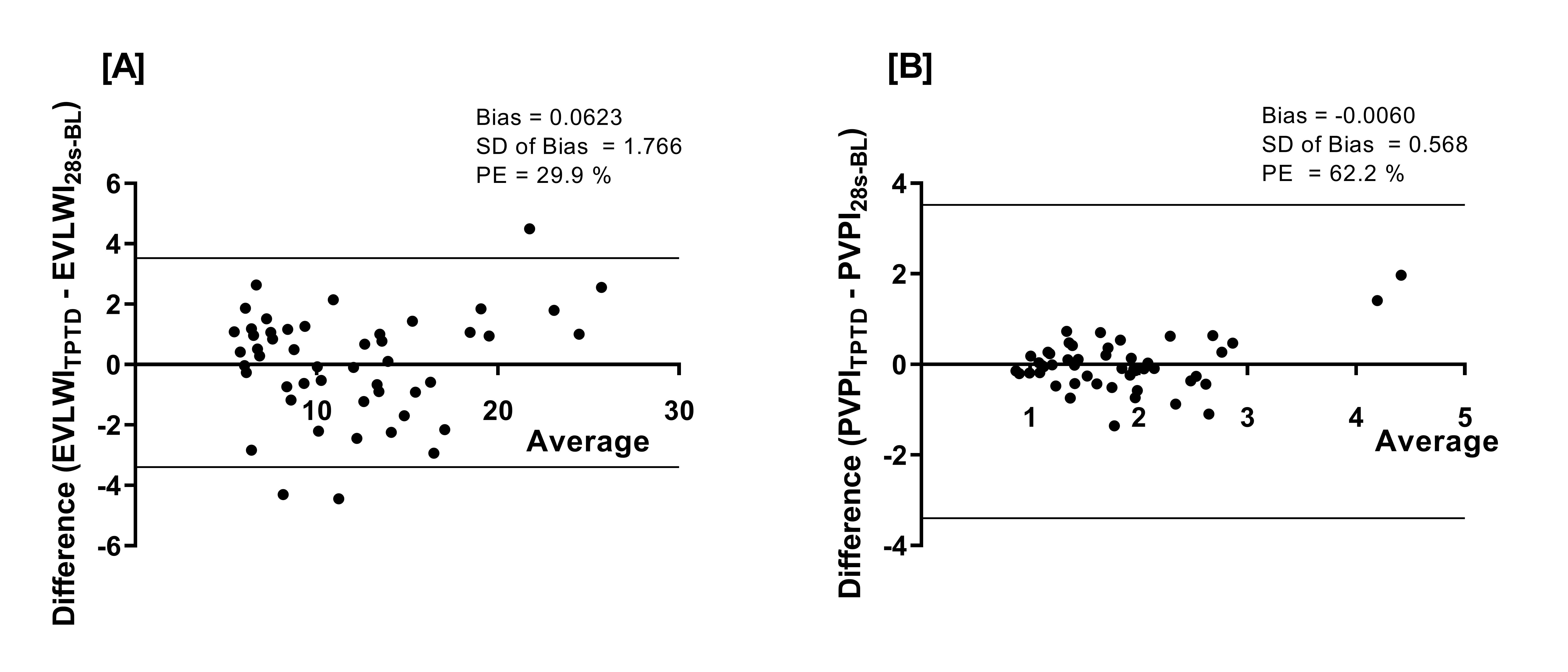

Supplement: Supplementary_file_3 - B-Lines Scores Derived From Lung Ultrasound Provide Accurate Prediction of Extravascular Lung Water Index: An Observational Study in Critically Ill Patients [file Supplementary_file_3.tif]

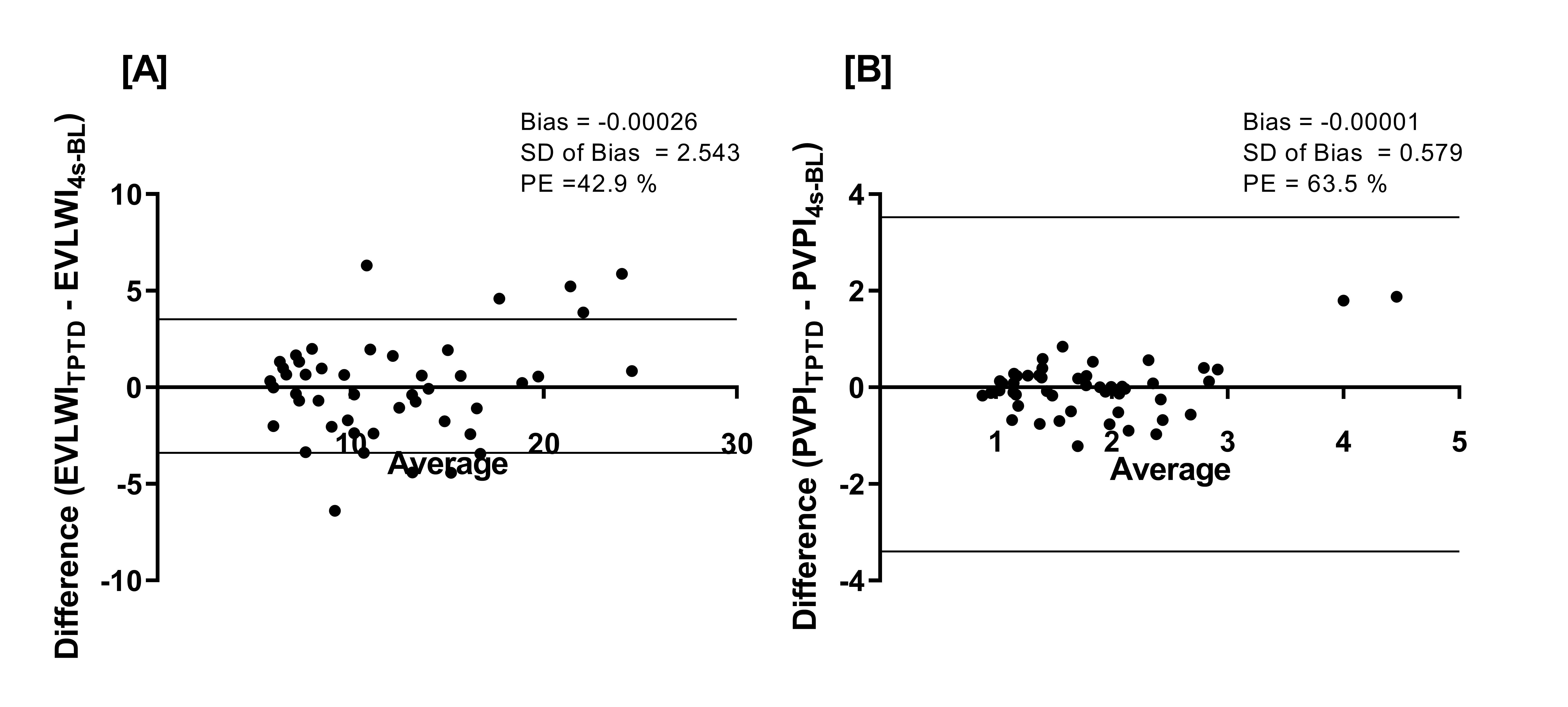

Supplement: Supplementary_file_4 - B-Lines Scores Derived From Lung Ultrasound Provide Accurate Prediction of Extravascular Lung Water Index: An Observational Study in Critically Ill Patients [file Supplementary_file_4.tif]
